# Genomic Characterization and Evolutionary Dynamics of SARS-CoV-2 Lineage NB.1.8.1 in Thailand, 2025

**DOI:** 10.3390/v18040450

**Published:** 2026-04-08

**Authors:** Jiratchaya Puenpa, Preeyaporn Vichaiwattana, Ratchadawan Aeemjinda, Sumeth Korkong, Ritthideach Yorsaeng, Yong Poovorawan

**Affiliations:** 1 Center of Excellence in Clinical Virology, Department of Pediatrics, Faculty of Medicine, Chulalongkorn University, Bangkok 10330, Thailand; jiratchaya.ph@chula.ac.th (J.P.); preeyaporn.vic@chulahospital.org (P.V.); aeemjinda.r@gmail.com (R.A.); sumeth.kor@gmail.com (S.K.); ritthideach.yor@gmail.com (R.Y.); 2 FRS(T), The Royal Society of Thailand, Sanam Sueapa, Dusit, Bangkok 10330, Thailand

**Keywords:** SARS-CoV-2, NB.1.8.1, Thailand, genomic surveillance, evolution dynamics

## Abstract

SARS-CoV-2 continues to cause recurrent waves in the post-pandemic period, yet genomic data from Southeast Asia remain limited for several emerging Omicron lineages, including NB.1.8.1. In this study, routine acute respiratory infection (ARI) surveillance performed in Bangkok, Thailand, from January to December 2025 was integrated with real-time RT-PCR testing and complete spike-gene sequencing for lineage assignment and evolutionary analysis. Among 4756 ARI specimens, 473 (9.9%) tested positive for SARS-CoV-2. Positivity increased in late April, peaked in May (epidemiological week 21; 58.4%), and declined through late June. Lineage typing was successful for 165/473 positive samples (34.9%), identifying 16 Pango lineages. Early 2025 showed heterogeneous circulation, including XEC- and XEC.8-related lineages, whereas NB.1.8.1 predominated during the main wave, accounting for 92.4% of typed cases in May and 89.8% in June. No recombination signals meeting predefined criteria were detected in the spike dataset. The mean spike substitution rate was estimated at 1.11 × 10^−3^ substitutions/site/year (95% HPD, 9.13 × 10^−4^–1.31 × 10^−3^), and the major Thai-containing NB.1.8.1 clade had an estimated tMRCA of 17 July 2024. These findings show that routine ARI surveillance combined with spike-based genomics can provide timely insights into SARS-CoV-2 circulation, lineage replacement, and ongoing viral evolution in Thailand.

## 1. Introduction

Coronavirus disease 2019 (COVID-19) remains a major global public health challenge, causing substantial morbidity and mortality, particularly among older adults and individuals with underlying medical conditions [1]. COVID-19 is caused by severe acute respiratory syndrome coronavirus 2 (SARS-CoV-2), a single-stranded, positive-sense RNA virus in the genus *Betacoronavirus* within the family *Coronaviridae*, first identified in late 2019 [2,3]. Its precise zoonotic origin and any intermediate host(s) remain unresolved [4]. Closely related sarbecoviruses have been identified in bats, and SARS-CoV-2-related coronaviruses have also been detected in Malayan pangolins [5,6]. Since its emergence, SARS-CoV-2 has become an established and ongoing global health problem. Reinfections have become increasingly common, driven by waning protection against infection over time and viral immune evasion, particularly among antigenically divergent variants [7,8,9].

As an RNA virus, SARS-CoV-2 continues to evolve, generating successive variants ranging from early lineages and pre-Omicron Variants of Concern (VOCs), such as Alpha, Beta, Gamma, and Delta, to Omicron and its various sublineages [10,11]. Within the Omicron era, this rapid diversification was most visible from late 2022 onward, when BA.2-derived recombinant XBB lineages (including XBB.1.5 and XBB.1.16) spread widely and dominated much of 2023, with some descendants still circulating in early 2024 [12,13]. The global variant landscape shifted again in late 2023 and early 2024, with BA.2.86-derived JN.1 rapidly becoming predominant, followed by the emergence of multiple JN.1 descendant lineages, including KP.2 and KP.3, and later recombinant variants such as XEC [14]. This continual replacement and growing diversity can reshape transmission patterns and complicate routine surveillance, making genomic data an important complement to case-based monitoring. In Thailand, these dynamics highlight the value of integrating weekly surveillance trends with time-resolved phylogenetic analyses to resolve lineage turnover and explore introduction patterns and the timing of local diversification.

In 2025, NB.1.8.1 emerged as a rapidly increasing descendant of the recombinant lineage XDV.1.5.1 and became predominant in Thailand during the study period [15]. As of 23 May 2025, WHO had documented NB.1.8.1 from the earliest sample collected on 22 January 2025 and designated the lineage as a Variant Under Monitoring (VUM) [15]. WHO assessments considered the overall public health risk low and anticipated continued effectiveness of available vaccines and antivirals, while noting that spike mutations may be associated with increased transmissibility and modest immune escape relative to LP.8.1 [15]. 

As of 21 February 2026, approximately 15,000 NB.1.8.1 sequences had been submitted to GISAID, indicating ongoing interest in its spread and regional evolutionary patterns [16]. However, genomic data from Thailand and Southeast Asia during this period remained limited, constraining the regional context needed to interpret NB.1.8.1 and the dynamics of contemporaneously circulating lineages. As of the same date, Thailand had submitted 279 NB.1.8.1 genomes to GISAID, representing approximately 4.4% of the 6403 NB.1.8.1 genomes reported from Asia, despite substantial local activity [16]. In particular, it remains unclear how rapidly NB.1.8.1 displaced co-circulating lineages in Thailand and whether its predominance reflected repeated introductions, local expansion, or both. In addition, the spike substitution patterns associated with NB.1.8.1 circulation in Thailand have not been well characterized.

To address these gaps, we generated and analyzed complete spike sequences from routine surveillance specimens collected in Thailand from January to December 2025. SARS-CoV-2-positive samples were identified by real-time RT-PCR, followed by spike-gene amplification and sequencing. We quantified monthly lineage composition, turnover, and typing coverage and reconstructed phylogenetic relationships using maximum-likelihood and time-scaled Bayesian maximum clade credibility (MCC) trees to identify Thai clusters and place local diversification in a global context. Together, these findings establish a genetic baseline for SARS-CoV-2 circulation in Thailand in 2025 and provide genomic context for ongoing epidemiological surveillance and interpretation of lineage turnover.

## 2. Materials and Methods

### 2.1. Ethics Statement

This study was approved by the Institutional Review Board of the Faculty of Medicine, Chulalongkorn University, Thailand (IRB No. 0977/67), and conducted in accordance with the Declaration of Helsinki. The Institutional Review Board waived the requirement for informed consent because the study involved de-identified retrospective data and no direct participant contact. Age and sex data were retrieved from medical records, and all analyses were conducted using anonymized datasets with no access to personally identifiable information by the study team.

### 2.2. Clinical Specimens and Molecular Testing

During January to December 2025, a total of 4756 nasopharyngeal samples were collected from patients presenting with acute respiratory illness (ARI) at King Chulalongkorn Memorial Hospital, Bangkok, Thailand. ARI was defined as fever (temperature ≥ 38 °C), cough, and symptom onset within the preceding 10 days. Respiratory specimens were collected in 1 mL of universal transport medium and submitted to the Center of Excellence in Clinical Virology, King Chulalongkorn Memorial Hospital, for routine respiratory virus surveillance. Specimens were tested using virus-specific real-time RT-PCR assays for severe acute respiratory syndrome coronavirus 2 (SARS-CoV-2), influenza A and B viruses, respiratory syncytial virus (RSV), parainfluenza virus types 1–4 (PIV1–4), human coronaviruses (HCoVs; HCoV-229E, HCoV-NL63, HCoV-OC43, and HCoV-HKU1), adenovirus, and rhinovirus.

Nucleic acids were extracted from 200 μL of sample supernatant using the magLEAD 12gC automated system (Precision System Science, Chiba, Japan) according to the manufacturer’s protocol. Detection of severe acute respiratory syndrome coronavirus 2 (SARS-CoV-2) was performed by real-time RT-PCR targeting the conserved N1 and N2 regions of the nucleocapsid gene, using published primers and probes as previously described [17]. The primer and probe sequences used for SARS-CoV-2 real-time RT-PCR are provided in Supplementary Table S1. Real-time RT-PCR was performed in a 20 µL reaction containing 3.0 µL of total RNA (100 ng to 1 µg), 10 µL of 2× SensiFAST Probe One-Step Mix (Meridian Bioscience, London, UK), 0.5 µM of each primer, 0.25 µM of each probe, 0.2 µL of reverse transcriptase, 0.4 µL of RiboSafe RNase Inhibitor, and RNase-free water to a final volume of 20 µL. Reactions were run on a LightCycler 480 real-time PCR system (Roche, Mannheim, Germany) with reverse transcription at 42 °C for 30 min, initial denaturation at 95 °C for 10 min, and 45 cycles of 95 °C for 15 s and 60 °C for 30 s. The N1 and N2 targets were monitored in the FAM and HEX channels, respectively.

Age and sex metadata were extracted from the medical records. Age was analyzed both continuously and by strata (0–4, 5–17, 18–49, 50–64, and ≥65 years). Weekly and monthly SARS-CoV-2 positivity were summarized descriptively, and age- and sex-based comparisons were performed as described in Section 2.5.

### 2.3. SARS-CoV-2 Viral Amplification

For lineage typing, SARS-CoV-2-positive specimens were randomly selected from the surveillance dataset. Samples with real-time RT-PCR cycle threshold (Ct) values ≤ 30 were selected for spike amplification and sequencing. Samples were excluded from lineage analysis if spike amplification or sequencing was unsuccessful. The complete SARS-CoV-2 spike gene was amplified by one-step RT-PCR using previously described protocols and primer sets [18]. Briefly, one-step RT-PCR was carried out in a 25 µL reaction containing 2–3 µL of total RNA (100 ng to 1 µg), 0.5 µM of each primer, 12.5 µL of 2× reaction mix (containing 0.4 mM each dNTP and 3.2 mM MgSO_4_), 1 µL of SuperScript III RT/Platinum Taq High Fidelity enzyme mix, and nuclease-free water. Amplification was performed using the SuperScript III One-Step RT-PCR System with Platinum Taq High Fidelity (Invitrogen, Carlsbad, CA, USA) according to the manufacturer’s instructions. The thermal cycling conditions consisted of reverse transcription at 45 °C for 30 min, followed by 40 cycles of denaturation at 95 °C for 30 s, annealing at 50 °C for 30 s, and extension at 68 °C for 1 min 45 s, with a final extension at 68 °C for 5 min. The annealing temperature was optimized through preliminary experiments, and amplification specificity was confirmed by agarose gel electrophoresis, which demonstrated a single band of the expected size (~1.1 kb) with no evidence of non-specific amplification. PCR products were sequenced bidirectionally using the same forward and reverse primers at First BASE Laboratories Sdn. Bhd. (Selangor Darul Ehsan, Malaysia).

### 2.4. Phylogenetic Reconstruction and Evolutionary Analysis

Phylogenetic and evolutionary analyses were conducted using a dataset of complete SARS-CoV-2 spike gene sequences, including newly generated Thai sequences and globally representative sequences retrieved from the GISAID database. Sequences in the final dataset were classified into putative Pango lineages using Pangolin v4.3 [19]. The temporal signal of the dataset was assessed in TempEst version 1.5.3 using root-to-tip regression to determine its suitability for subsequent time-scaled phylogenetic inference [20]. Maximum-likelihood (ML) and maximum clade credibility (MCC) trees were rooted with BA.2.86 and representative early JN.1 lineages (including JN.1 and JN.1.16), which served as reference lineages for temporal interpretation of the 2025 dataset. Genome sequences were aligned using MAFFT v7.310 [21]. Maximum-likelihood phylogenetic trees were reconstructed in MEGA X (v10.2.6) [22], and branch support was evaluated with 1000 bootstrap replicates. The optimal nucleotide substitution model was identified in MEGA X using the Find Best DNA/Protein Models (ML) option according to the lowest BIC score.

Time-scaled Bayesian phylogenetic analysis was conducted in BEAST v1.10.4 using a strict molecular clock and a constant population size, as previously described [18,23]. Markov chain Monte Carlo (MCMC) chains were run for 400 million steps, with sampling performed every 40,000 generations. Convergence and mixing were evaluated in Tracer v1.7.1 [24], and effective sample size (ESS) values exceeded 200 for all key parameters after discarding the first 10% of samples as burn-in. Maximum clade credibility (MCC) trees were generated from the posterior tree distribution using TreeAnnotator v1.8.4 and visualized in FigTree (https://github.com/rambaut/figtree/releases (accessed on 14 February 2026)).

Amino-acid substitutions across the spike (S) protein were analyzed relative to the Wuhan-Hu-1 reference sequence (NC_045512.2) to identify lineage-specific substitution patterns. For each lineage, the prevalence of substitution at each amino-acid position was calculated using sequences with valid calls. Positions with substitution prevalence greater than 50% and at least 10 valid observations (n_valid ≥ 10) were selected for downstream visualization in R (version 4.4.2) [25].

To investigate possible recombination, the complete spike sequence dataset included in the phylogenetic analyses (*n* = 449) was examined using RDP5 [26]. The dataset comprised study-derived sequences together with globally available SARS-CoV-2 reference sequences downloaded from the NCBI GenBank database and aligned prior to analysis. Evidence for recombination was explored using eight analytical approaches implemented in RDP5, including RDP, GENECONV, BootScan, MaxChi, Chimaera, SiScan, 3Seq, and LARD. Only events supported by at least five methods at a significance threshold of *p* < 0.05 were retained as potential recombination signals, following conservative criteria used for SARS-CoV-2 related coronavirus studies [27].

### 2.5. Statistical Analysis

Statistical analyses for epidemiologic comparisons were performed in R (version 4.4.2) [25]. Differences in SARS-CoV-2 positivity across age strata were evaluated using logistic regression to estimate odds ratios with 95% confidence intervals, using adults aged 18–49 years as the reference group. Continuous age distributions between lineage groups were compared using a two-sided Wilcoxon rank-sum test, with effect sizes summarized using Cliff’s delta and the Hodges–Lehmann location shift with 95% confidence intervals. Sex distributions were compared using Fisher’s exact test. Unless otherwise specified, tests were two-sided and *p* < 0.05 was considered statistically significant, and analyses were restricted to records with non-missing values for the corresponding variables.

For site-specific selection, Hypothesis Testing Using Phylogenies (HyPhy) v2.4.0 was used to infer site-specific selection using Fixed Effects Likelihood (FEL), the Mixed Effects Model of Evolution (MEME), Single-Likelihood Ancestor Counting (SLAC), and Fast Unconstrained Bayesian AppRoximation (FUBAR) [28]. Evidence for episodic diversifying selection was assessed with MEME, considering sites with *p* < 0.1 as supported. Evidence for pervasive positive selection was assessed with FUBAR, considering sites with posterior probability PP ≥ 0.95 as supported. For additional frequentist site models, statistically significant positively selected sites were defined as *p* < 0.05 for FEL and *p* < 0.1 for SLAC.

## 3. Results

### 3.1. SARS-CoV-2 Positivity and Demographic Characteristics in ARI Surveillance

**Figure 1:**
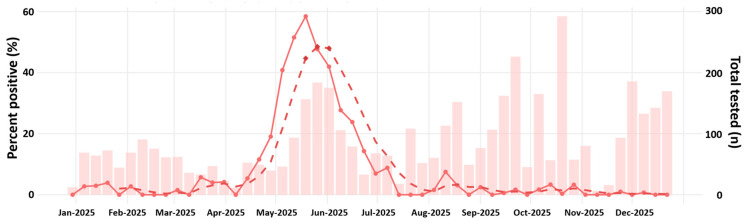
Weekly SARS-CoV-2 positivity in Thailand in 2025. Weekly percent positivity is shown by the solid line with points (left *y*-axis), and the 5-week weighted moving average (WMA5) is shown by the dashed line. Bars indicate the total number of ARI specimens tested each week (right *y*-axis). Weeks corresponding to the highest 5% of WMA5 values are highlighted as peak activity.

**Figure 2:**
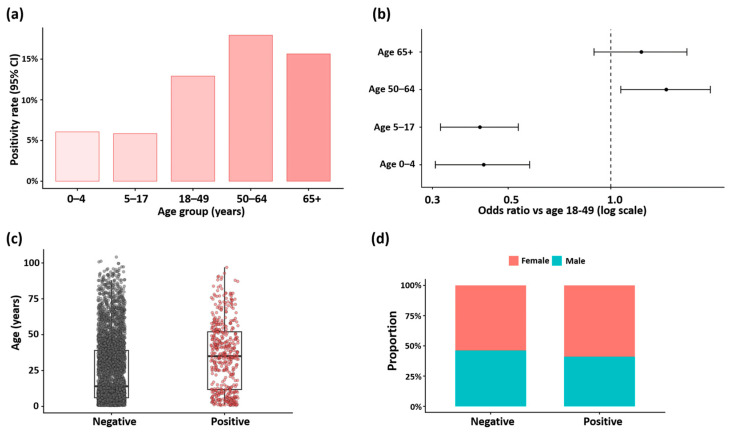
Age and sex distributions of SARS-CoV-2 test results and age-stratified positivity. (**a**) SARS-CoV-2 positivity rate by age group. (**b**) Odds ratios for SARS-CoV-2 positivity by age group relative to adults aged 18–49 years (reference), shown on a log scale with 95% confidence intervals; the dashed vertical line indicates an odds ratio of 1.0. (**c**) Age distribution by SARS-CoV-2 test result. (**d**) Sex distribution by SARS-CoV-2 test result.

To describe seasonal variation in SARS-CoV-2 activity, we tested 4756 ARI specimens for SARS-CoV-2 by real-time RT-PCR during January–December 2025 and summarized weekly positivity over time (Figure 1). Overall, 473/4756 specimens were SARS-CoV-2-positive (9.9%). Weekly positivity showed a single rise beginning in late April, reaching a maximum in epidemiological week 21 (May; weekly positivity 58.4%), and declining through the end of June (weekly positivity 6.9%) (Figure 1). Positivity increased with age, peaking among adults aged 50–64 years, while remaining lower in children and adolescents (Figure 2a). Using adults aged 18–49 years as the reference group, the odds of SARS-CoV-2 positivity were lower in children aged 0–4 years (OR 0.42, 95% CI 0.31–0.58; *p* < 0.001) and 5–17 years (OR 0.41, 95% CI 0.32–0.54; *p* < 0.001), but higher in adults aged 50–64 years (OR 1.45, 95% CI 1.07–1.96; *p* = 0.015) (Figure 2a). For adults aged ≥ 65 years, the odds were not significantly different from the reference group (OR 1.23, 95% CI 0.89–1.67; *p* = 0.197) (Figure 2b). Age also differed by test result: positive specimens were older than negative specimens (positive: median 35.0 years [IQR 11.8–52.0], *n* = 464; negative: median 14.0 years [IQR 6.0–39.0], *n* = 4265; Wilcoxon rank-sum test, *p* < 0.001) (Figure 2c). Sex distributions did not differ significantly between SARS-CoV-2-positive and SARS-CoV-2-negative specimens (Figure 2d; Fisher’s exact test, *p* > 0.05). Age-stratified analyses were restricted to specimens with non-missing age metadata, accounting for the reduced sample sizes relative to the full tested cohort.

### 3.2. Lineage Typing Coverage Among SARS-CoV-2-Positive Specimens

**Figure 3:**
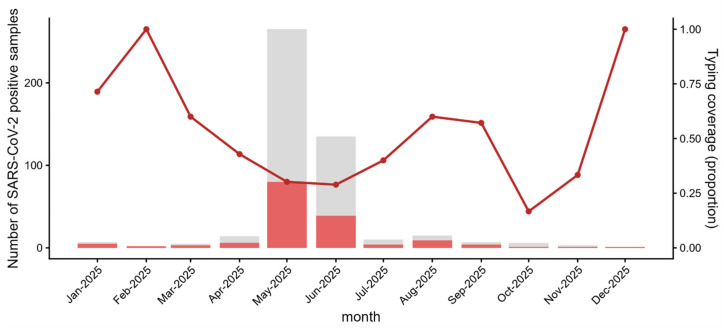
Monthly typing coverage among SARS-CoV-2-positive specimens. Grey bars indicate the total number of SARS-CoV-2-positive samples collected each month, and red bars indicate the number with successful lineage typing. The line (right *y*-axis) shows monthly typing coverage, defined as the proportion typed (typed/positive). Months with very small numbers of positives may yield unstable coverage estimates and should be interpreted cautiously. Lineage typing was performed on a randomly selected subset of positive specimens.

Among 473 SARS-CoV-2-positive specimens collected in 2025, 165 were successfully lineage-typed (overall coverage 34.9%). The highest monthly number of positives occurred in May 2025 (266 positives), with 79 typed (29.7%), followed by June 2025 (136 positives, 49 typed, 36.0%). Typing coverage tended to be higher in months with fewer positives, reaching 66.7% in August 2025 (10/15) and 42.9% in April 2025 (6/14). The lowest monthly coverage was observed in October 2025 (1/6, 16.7%). Because the October estimate was based on only one typed sample, it was too small to represent the monthly distribution and should be interpreted with caution. Months with very small numbers of positives (e.g., February 2025: 2/2; December 2025: 1/1) yielded unstable proportions and should be interpreted cautiously (Figure 3). Accordingly, lineage composition and turnover were interpreted with greatest confidence during months with larger numbers of typed specimens (May–June).

### 3.3. Temporal Lineage Turnover and Predominance of NB.1.8.1 in 2025

**Figure 4:**
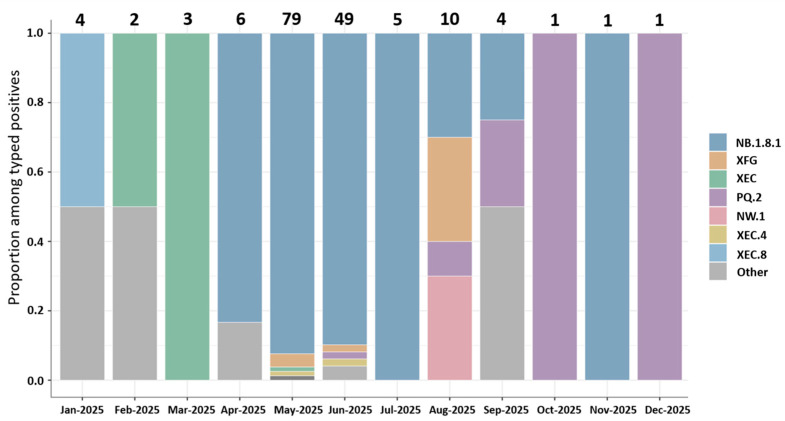
SARS-CoV-2 lineage composition by month.

Among typed SARS-CoV-2-positive specimens, 16 distinct Pango lineages were identified (Figure S1). NB.1.8.1 dominated the mid-year period, rising to 83.3% in April (*n* = 6) and accounting for the large majority of typed positives during the peak months of May (92.4%, *n* = 79) and June (89.8%, *n* = 49), with continued predominance in July (100%, *n* = 5) (Figure 4). In contrast, early 2025 showed greater heterogeneity with XEC- and XEC.8-related lineages, whereas later months included increased representation of PQ.2. For September–December, month-specific proportions are based on very small numbers of typed specimens and are therefore imprecise.

**Figure 5:**
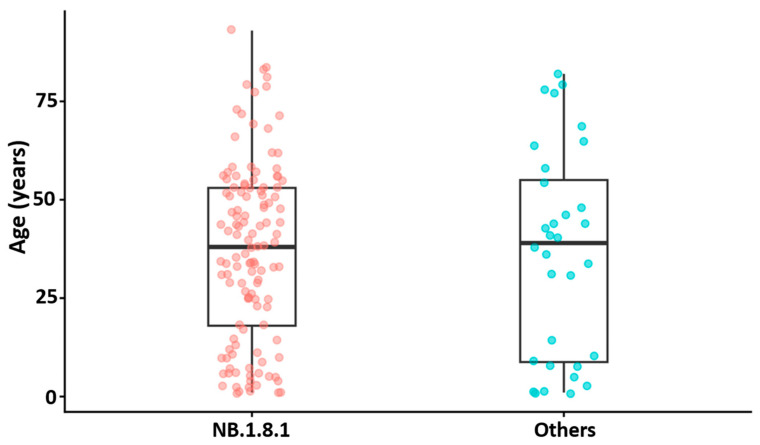
Age distribution by lineage group. Boxplots summarize age for NB.1.8.1 versus other lineages (Others). Boxes indicate median and IQR, whiskers 1.5 × IQR, and points represent individual samples. *p*-value from a two-sided Wilcoxon rank-sum test.

Age distributions were similar between NB.1.8.1 and other lineages, with overlapping medians and IQRs (Figure 5). There was no evidence of an age difference between groups by Wilcoxon rank-sum testing (*p* = 0.834), and the estimated location shift was small (Hodges–Lehmann estimate = 1.0 year; 95% CI −9.0 to 10.0). The corresponding effect size was negligible (Cliff’s δ = 0.02; 95% CI −0.22 to 0.26). Sex proportions were comparable between NB.1.8.1 and other lineages (Fisher’s exact test, *p* = 1.00).

### 3.4. Phylogenetic Structure and Evolutionary Dynamics of Thai SARS-CoV-2 Lineages

**Figure 6:**
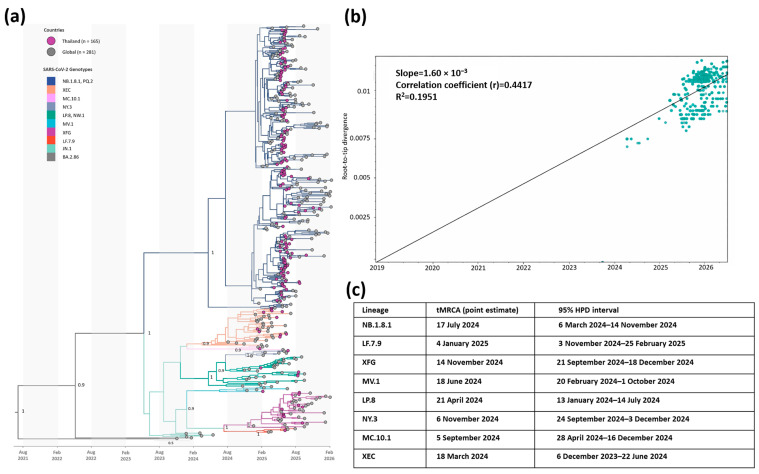
Time-scaled phylogenetic and temporal analyses of SARS-CoV-2 spike sequences derived from Thailand in 2025. (**a**) Maximum clade credibility (MCC) tree inferred under a molecular clock framework from complete spike sequences obtained in this study from Thailand (*n* = 165) together with global reference sequences (*n* = 281). The *x*-axis indicates calendar time, and the scale bar represents substitutions per site. Tips are colored according to sampling location, branches are colored by major Pango lineage group, and selected internal nodes are annotated with posterior probability (PP). (**b**) Root-to-tip regression of genetic divergence against sampling time, showing the temporal signal of the spike dataset used for molecular clock analysis. (**c**) Estimated times to the most recent common ancestor (tMRCA) and corresponding 95% highest posterior density (HPD) intervals for the major lineage groups identified in the MCC tree.

No recombination signals meeting our predefined RDP5 threshold were detected in the spike alignment analyzed in this study. The alignment was therefore used for downstream phylogenetic and molecular clock analyses. To investigate the evolutionary relationships between SARS-CoV-2 sequences from Thailand and global reference datasets, we reconstructed a time-scaled maximum clade credibility (MCC) phylogeny under a Bayesian molecular clock framework (Figure 6). The mean spike substitution rate was 1.11 × 10^−3^ nucleotide substitutions per site per year (s/s/y), with a 95% highest posterior density (HPD) interval of 9.13 × 10^−4^–1.31 × 10^−3^. As an exploratory assessment of temporal signal, root-to-tip regression in TempEst indicated only a modest clock-like pattern (r = 0.44, R^2^ = 0.20) and an approximate slope-based rate of 1.60 × 10^−3^ s/s/y; these results were interpreted alongside, but not used directly to derive, the Bayesian molecular clock estimates.

Consistent with the main epidemic wave occurring during May–July 2025, 133 of 165 Thai genomes (80.6%) contributing to the MCC phylogeny were sampled within this period, reflecting both intensified transmission and increased sequencing during the peak. Within NB.1.8.1, a strongly supported internal node (posterior probability [PP] = 1.0) defined a minor subclade comprising only global sequences (Taiwan, *n* = 2; Malaysia, *n* = 1; Switzerland, *n* = 1), with no Thai sequences observed. In contrast, the main NB.1.8.1 clade contained 124 Thai and 152 global sequences, with Thai genomes interspersed among global references. Although deeper relationships within this clade were only moderately supported (PP = 0.80), the limited geographic segregation suggests weak phylogeographic structure at this resolution, acknowledging potential constraints from available sampling. The tMRCA of the major Thai-containing NB.1.8.1 clade was estimated as 17 July 2024 (95% HPD, 6 March 2024–14 November 2024).

In addition to NB.1.8.1, sequences assigned to other co-circulating lineages (including XEC, LP.8/NW.1, XFG, MV.1, MC.10.1, NY.3, and LF.7.9) were resolved as lineage-specific clades in the MCC phylogeny, with key internal nodes generally supported at PP ≥ 0.9. These lineages were represented by smaller clusters than NB.1.8.1 and were sampled predominantly during 2025. Depending on the lineage, Thai genomes were either interspersed among global references or formed one or more Thai-enriched subclusters at the tips, consistent with limited geographic segregation at this phylogenetic resolution.

Estimated tMRCAs for non-NB.1.8.1 lineages spanned the period from late 2023 to early 2025, indicating staggered lineage origins underlying the diversity observed during the 2025 sampling window. The earliest inferred origin was observed for XEC (tMRCA 18 March 2024, 95% HPD 6 December 2023–22 June 2024), followed by LP.8 (tMRCA 21 April 2024, 95% HPD 13 January–14 July 2024) and MV.1 (tMRCA 18 June 2024, 95% HPD 20 February–1 October 2024). Several additional lineages exhibited mid-to-late-2024 origins, including MC.10.1 (tMRCA 5 September 2024, 95% HPD 28 April–16 December 2024), NY.3 (tMRCA 6 November 2024, 95% HPD 24 September–3 December 2024), and XFG (tMRCA 14 November 2024, 95% HPD 21 September–18 December 2024). In contrast, LF.7.9 showed a more recent tMRCA (4 January 2025, 95% HPD 3 November 2024–25 February 2025), consistent with comparatively late emergence within the study period.

### 3.5. Spike Variation Patterns and Signatures of Positive Selection

**Figure 7:**
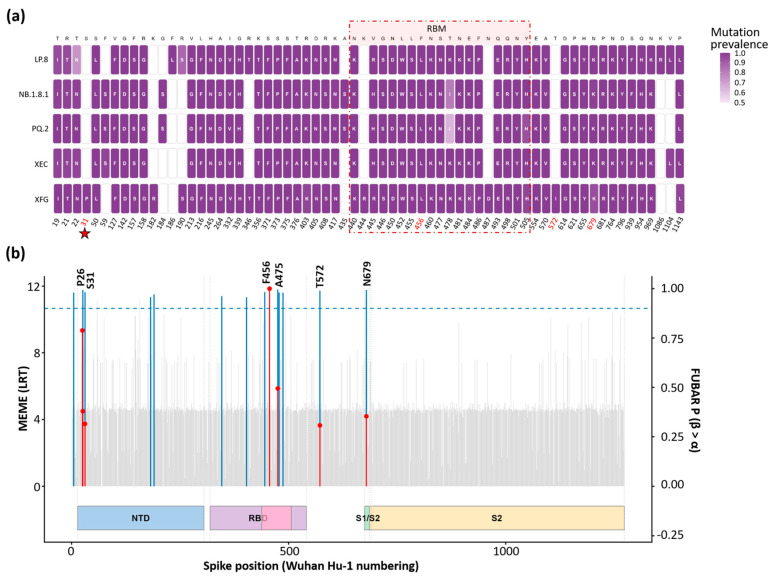
Spike amino-acid variation and site-specific positive selection in SARS-CoV-2. (**a**) Amino-acid variation landscape of the SARS-CoV-2 spike protein across major lineages. Amino-acid changes were determined relative to the Wuhan-Hu-1 reference sequence (NC_045512.2). Tile color indicates the prevalence of each amino-acid change at a given spike position within each lineage. Only positions with change prevalence > 50% among sequences with valid amino-acid calls (n_valid ≥ 10) are displayed. Red position numbers indicate sites with evidence of positive selection. The red asterisk at spike position 31 indicates that variation at this site included both deletion and substitution states across different lineages. (**b**) Overlay of site-wise evidence for episodic and pervasive positive selection across the SARS-CoV-2 spike protein (Wuhan-Hu-1 numbering). Red lollipops show MEME likelihood ratio test (LRT) statistics for episodic diversifying selection; red points denote significant sites (*p* < 0.1). Vertical bars represent FUBAR posterior probabilities (PP) of pervasive positive selection (right *y*-axis), with sites exceeding the significance threshold (PP ≥ 0.95) highlighted in blue. Dashed horizontal lines indicate the corresponding significance cutoffs. Spike domain boundaries (NTD, RBD/RBM, S1/S2, and S2) are shown schematically along the *x*-axis. Labeled positions indicate sites meeting the significance criteria in at least one method.

To identify key substitution sites, we quantified lineage-stratified variation across the spike (S) protein relative to Wuhan-Hu-1 and visualized positions with change prevalence > 50% among sequences with valid calls (n_valid ≥ 10) (Figure 7a). Across lineages, a consistent set of high-prevalence changes was observed, with several sites approaching fixation (prevalence near 1.0). Notably, variation at spike position 31 included both deletion and substitution states across different lineages, whereas other high-prevalence sites were predominantly represented by amino-acid substitutions. Among the 66 high-prevalence sites, two substitutions within the receptor-binding motif (RBM; 438–506), at positions 435 and 478, were observed in NB.1.8.1 and were retained in the subsequently detected lineage PQ.2. In addition, NB.1.8.1 harbored a high-prevalence substitution at position 188 (G184S), located in the S1 N-terminal domain (NTD), a region enriched for antibody-targeted epitopes, indicating that prominent changes were not confined to the RBM. LP.8 and its descendant lineages also exhibited high-prevalence substitutions in the NTD, including F186L and R190S.

Because several high-prevalence changes were mapped to, or adjacent to, known antigenic regions of spike, we next assessed evidence of positive selection using MEME (episodic) and FUBAR (pervasive) analyses (Figure 7b). Sites with MEME *p* < 0.1 and/or FUBAR posterior probability (PP) ≥ 0.95 were considered supported. Supported sites were confined to S1, clustering in the NTD and RBD/RBM and near the S1/S2 junction, whereas no sites in S2 met the predefined significance thresholds (Figure 7b). Within S1, P26 and S31 showed strong signals of episodic diversifying selection in the NTD, and two RBD sites (F456 and A475) also remained supported by MEME, indicating ongoing episodic positive selection in this antigenically important region. T572, located in S1 (subdomain 1), was supported by both MEME and FUBAR and was additionally identified by SLAC (*p* < 0.1) (Supplementary Table S2). Moreover, N679, which lies adjacent to the S1/S2 cleavage site, was also supported by MEME as a site under episodic diversifying selection. Concordance analyses using additional site models further supported a subset of these signals (Supplementary Table S2). Specifically, FEL (*p* < 0.05) also identified sites 26, 475, and 679, whereas SLAC (*p* < 0.1) supported site 572. These site-specific signals should be interpreted as statistical evidence suggestive of selection rather than definitive proof of functional adaptation.

## 4. Discussion

In this study, we use routine surveillance to characterize the epidemiological and evolutionary features of SARS-CoV-2 in Thailand during 2025. We describe the epidemiological profile of acute respiratory infection (ARI) associated with SARS-CoV-2 and generate 165 complete spike sequences from PCR-confirmed specimens collected throughout the year. These findings provide a baseline genomic snapshot of SARS-CoV-2 circulation in Thailand in 2025 and support interpretation of lineage turnover in the post-pandemic period.

The SARS-CoV-2 positivity in our ARI surveillance (9.9%) was broadly consistent with post-pandemic sentinel respiratory surveillance reported through GISRS to FluNet, with WHO reporting sentinel positivity of ~8% globally and ~11% in the WHO South-East Asia Region [29]. While direct comparisons should be interpreted cautiously due to differences in case definitions and surveillance designs, our positivity was higher than estimates reported in some other post-pandemic respiratory surveillance settings, including Ethiopia and Bangladesh [30,31]. The age gradient observed in 2025 suggests that mid-life adults (50–64 years) contributed substantially to symptomatic ARI burden during the study period, and we found no evidence of sex-based differences in positivity. Several studies have reported sex-associated differences in SARS-CoV-2 test positivity or case detection, including higher odds of a positive test among men in a UK primary-care sentinel network [31,32]. Similar patterns have also been observed in primary-care sentinel surveillance, where middle-aged adults (40–64 years) showed the greatest risk of testing positive compared with children [32].

Our center-based ARI surveillance showed that the seasonal peak of SARS-CoV-2 did not occur at the same time each year. In 2025, positivity reached its highest level in June, whereas the corresponding peak in 2024 occurred in April on the same surveillance platform [18]. Although this difference was modest, it suggests that SARS-CoV-2 activity in Thailand may not follow a fixed seasonal pattern. The June 2025 peak occurred during the early rainy season, which could have influenced transmission through changes in human behavior, including increased time spent indoors. This contrasts with observations from temperate regions, where SARS-CoV-2 activity has more often been concentrated in winter [33,34]. A similar lack of synchrony was seen in Thai multi-pathogen ARI surveillance in 2024, in which SARS-CoV-2 peaked earlier than influenza rather than rising in parallel [35]. These observations support the value of year-round surveillance to capture shifts in peak timing and to contextualize changes in circulating lineages in the post-pandemic period.

Consistent with this pattern, lineage composition changed over the course of 2025. XEC and its descendants were detected mainly during January–March, whereas NB.1.8.1 increased from May and peaked in June, becoming the dominant lineage during the main 2025 peak in our data. This rise was also consistent with contemporaneous global reports: by mid-May 2025, NB.1.8.1 sequences had been submitted to GISAID from over 20 countries [36]. NB.1.8.1 was subsequently reported by ECDC as one of the variants circulating at low proportions in the EU/EEA in June 2025 [37]. To place these lineage dynamics in an evolutionary context, we estimated a mean spike-gene substitution rate of 1.11 × 10^−3^ substitutions per site per year. This estimate is comparable to substitution rates reported for Omicron-era SARS-CoV-2 in other settings, which are typically on the order of 10^−3^ substitutions per site per year [38,39].

In the current study, all six positively selected sites identified in the spike protein (P26, S31, F456, A475, T572, and N679) were located within the S1 subunit, spanning the N-terminal domain (NTD), the receptor-binding domain/receptor-binding motif (RBD/RBM), and the region proximal to the S1/S2 junction. Our findings are consistent with previous reports showing that adaptive evolution in the SARS-CoV-2 spike is concentrated in S1 rather than S2. For example, earlier LRT-based analyses of a global early-pandemic spike dataset (including VOC backgrounds such as Alpha, Beta, Gamma, and Delta-related lineages) identified at least 51 positively selected sites, including P26 and T572, which were also detected in our dataset supporting the view that these positions may represent recurrent targets of adaptive change [40]. In our Thai sequences, variation at spike position 31 was observed across multiple lineages and included both deletion and substitution states. Among these, S31del was detected in several lineages, including MC.10.1, LP.8.1.6, MV.1, NW.1, and NY.3, consistent with repeated emergence of this NTD change in recent viral backgrounds. Recent structural and evolutionary analyses of KP.3.1.1 indicate that S31del introduces a new N30 glycosylation site and is accompanied by local NTD rearrangement including an F32 side chain flip, while re occurring mutations such as S31del and F456L appear to have become more frequent since late 2023 [41,42]. Nonetheless, site-based selection inference does not establish phenotypic impact, and functional studies will be required to clarify the mechanistic consequences of these substitutions.

This study has several limitations. First, we generated spike-only sequences and did not perform whole-genome sequencing. While complete spike sequences are informative for lineage assignment and time-resolved phylogenetic inference, whole-genome data would provide a higher resolution for detecting recombination, resolving fine-scale transmission links, and characterizing mutations outside spike. Second, the samples analyzed in this study were collected from only one province. Since Thailand consists of 77 provinces, the findings may not be fully representative of the national population. Third, clinical data were not available for the patients included in this study. As a result, we were unable to evaluate the association between viral lineages and disease severity. Fourth, we did not incorporate population immunity data, such as vaccination coverage, booster uptake, or prior infection history, which may have influenced lineage turnover and transmission dynamics during the study period. Fifth, lineage typing was successful for only a subset of SARS-CoV-2-positive specimens, and although samples were randomly selected, incomplete typing coverage may have introduced uncertainty in lineage representation, particularly in months with small numbers of positive or typed specimens. Sixth, the spike dataset showed only modest temporal signal in root-to-tip regression, which may increase uncertainty in time-scaled estimates such as substitution rates and tMRCAs. Future work integrating richer clinical information with broader genomic surveillance, together with functional studies such as neutralization assays and complementary structural analyses, will be important to better understand genotype–phenotype relationships and the antigenic consequences of spike variation in the post-pandemic period.

In conclusion, our findings show that SARS-CoV-2 lineage dynamics in Thailand remained highly fluid during 2025, with ongoing turnover among circulating variants. Our findings, together with previous reports, indicate that the circulating SARS-CoV-2 lineages continue to change over time. This study provides a useful reference for understanding the possible evolutionary trajectories of SARS-CoV-2 and highlights the likelihood that new variants will continue to emerge.

## Data Availability

Sequences generated from this study were deposited in the NCBI GenBank database under accession numbers PZ120506–PZ120670.

## Supplementary Materials

The following supporting information can be downloaded at https://www.mdpi.com/article/10.3390/v18040450/s1.
