# Supplementary material for: Genomic Characterization and Evolutionary Dynamics of SARS-CoV-2 Lineage NB.1.8.1 in Thailand, 2025
Source: Viruses. 2026 Apr 8;18(4):450. doi: 10.3390/v18040450 (PMC13120155; doi:10.3390/v18040450)
Supplement: Supplementary file 1 [file viruses-18-00450-s001.zip › viruses-4225606-supplementary.pdf]

Genomic characterization and evolutionary dynamics of SARS-CoV-2 lineage NB.1.8.1 in Thailand, 2025

Jiratchaya Puenpa[1], Preeyaporn Vichaiwattana[1], Ratchadawan Aeemjinda[1], Sumeth Korkong[1], Ritthideach Yorsaeng[1], and Yong Poovorawan[1,2*]

Table S1. Primer and probe sequences used for SARS-CoV-2 real-time RT-PCR

**Name**	**Nucleotide sequence 5´- 3´**	**Target gene**	**Product size**
N1_F	GACCCCAAAATCAGCGAAAT	Nucleocapsid gene	73 bp
N1_R	TCTGGTTACTGCCAGTTGAATCTG		
N1_Probe	ACCCCGCATTACGTTTGGTGGACC		
N2_F	TTACAAACATTGGCCGCAAA		67 bp
N2_R	GCGCGACATTCCGAAGAA		
N2_Probe	ACAATTTGCCCCCAGCGCTTCAG		

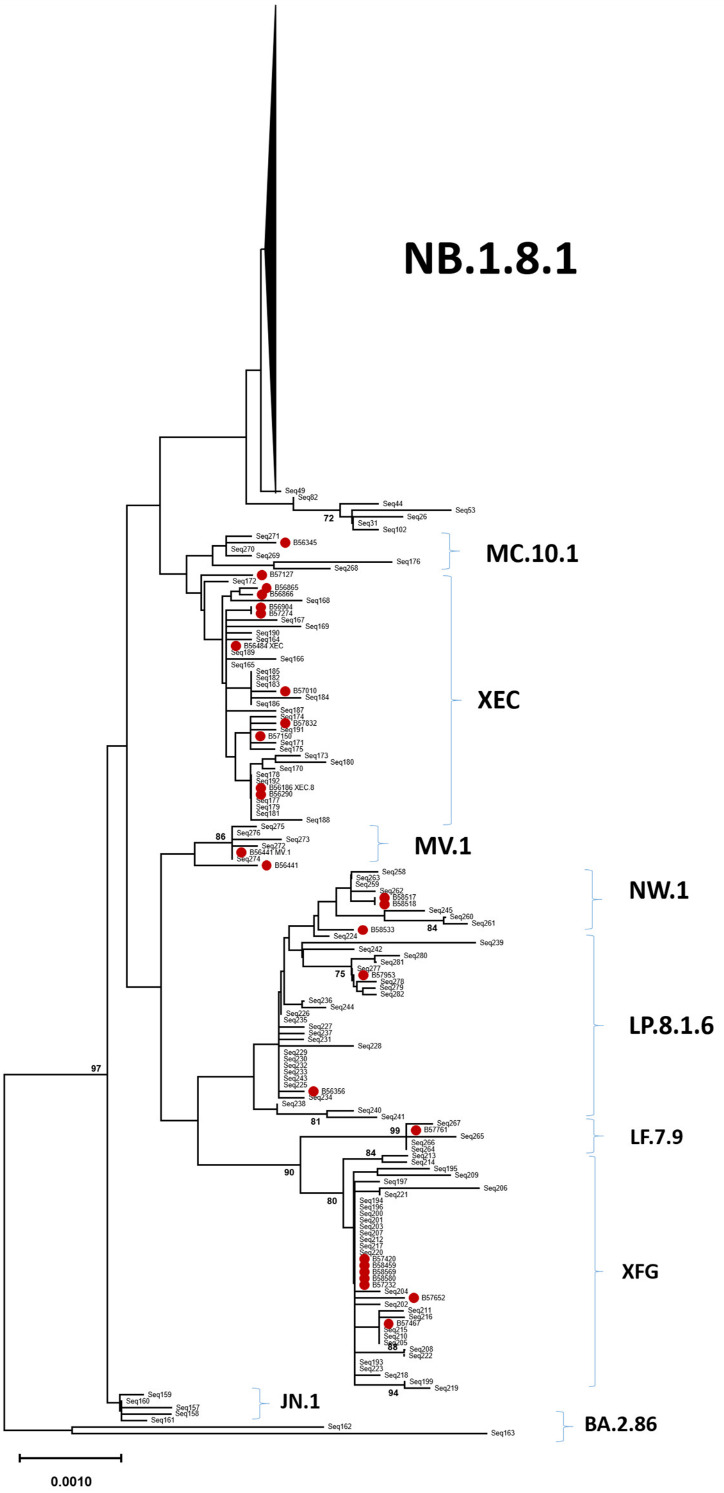

Figure S1. Maximum-likelihood phylogeny of SARS-CoV-2 spike sequences from Thailand in 2025.

The maximum-likelihood (ML) tree was reconstructed from spike gene sequences using IQ-TREE2 with

1,000 ultrafast bootstrap replicates. Bootstrap support values ≥70% are shown at the corresponding nodes. Major lineages/clades (NB.1.8.1, MC.10.1, XEC, MV.1, NW.1, LP.8.1.6, LF.7.9, XFG, JN.1, and BA.2.86) are indicated by brackets. Thai sequences generated in this study are highlighted in red. The scale bar indicates substitutions per site.

Table S2. Summary of site-specific selection analyses in the SARS-CoV-2 spike protein using FEL, SLAC, FUBAR, and MEME.

Method	Criterion	Positive sites (Wuhan Hu-1)	Negative sites (Wuhan Hu-1)
FEL	$p < 0.05$	26, 475, 679	11, 106, 225, 412, 460, 500, 778, 852, 856, 872, 938, 1071, 1110, 1122, 1148, 1181, 1195, 1215, 1263
SLAC	$p < 0.1$	572	25, 106, 336, 354, 410, 452, 543, 778, 824, 856, 938, 1110, 1148, 1215
FUBAR	$PP \geq 0.95$	5, 26, 31, 182, 190, 346, 403, 445, 456, 475, 478, 487, 572, 679	25, 106, 336, 500, 543, 682, 778, 821, 856, 938, 1110, 1122, 1148, 1215
MEME	$p < 0.1$	25, 26, 31, 456, 475, 572, 679	N/A
